# Virtual reality training for gait disorders in Parkinson’s disease: a bibliometric and visual analysis of hotspots and trends using CiteSpace and VOSviewer

**DOI:** 10.3389/fneur.2026.1848878

**Published:** 2026-06-11

**Authors:** Yutong Pan, Yiyang Huang, Mingming Bao, Xiandong Sun

**Affiliations:** 1 School of Occupational Therapy, Massachusetts College of Pharmacy and Health Sciences, Worcester, MA, United States; 2 Rehabilitation Therapy Program, Clinical Medical College of Tianjin Medical University, Tianjin, China; 3 Department of Rehabilitation Medicine, Chifeng Municipal Hospital, Chifeng, Inner Mongolia, China; 4 Department of Hypertension Center, Chifeng Municipal Hospital, Chifeng, Inner Mongolia, China

**Keywords:** gait disorders, neurologic, Parkinson disease, rehabilitation, virtual reality

## Abstract

**Background:**

This study systematically reviews the current status, hotspot distribution, and developmental trends of virtual reality (VR) training for improving gait disorders in Parkinson’s disease (PD).

**Aim:**

To map the global intellectual landscape and emerging frontiers of VR training for PD gait disorders (primary objective), while identifying developmental gaps in Chinese research through cross-database comparative analysis (secondary objective).

**Methods:**

Relevant literature published between 2008 and 2025 was retrieved from the Web of Science, PubMed, and CNKI databases. Ultimately, 535 English and 18 Chinese articles were included. CiteSpace and VOSviewer were used to conduct visual analyses of publication volume, cooperation networks (countries, authors, and institutions), keyword co-occurrence, clustering, and burst terms.

**Results:**

English publication volume grew steadily, peaking between 2020 and 2025, whereas Chinese literature yielded only 18 articles (averaging fewer than three annually). The United States (108 papers) occupies the core position in the national cooperation network. China ranks third in publication volume (42 papers) but lacks extensive international collaboration. English keywords focused on “virtual reality,” “gait,” “balance,” and “augmented reality,” while Chinese keywords centered on similar core concepts. Burst term analysis indicates that research frontiers have shifted from “physical therapy” and “neuroplasticity” toward intelligent, remote, and personalized directions, such as “wearable sensors,” “telerehabilitation,” and “deep learning.”

**Discussion:**

VR training for PD gait disorders represents an expanding global research domain. Under strictly applied database parameters, cross-linguistic analysis reveals distinct thematic focuses rather than a baseline disparity. International English literature predominantly addresses technical frontier iterations, while Chinese-language publications demonstrate a highly specialized path dedicated to localized clinical application and efficacy verification. Future studies should strengthen multi-center cross-linguistic collaboration, emphasize precision rehabilitation and neural mechanisms, and promote personalized telerehabilitation interventions.

## 1 Introduction

Parkinson’s disease (PD) is the second most prevalent neurodegenerative disorder worldwide, surpassed only by Alzheimer’s disease, with a prevalence of approximately 1% among individuals aged over 60 years (1). As global population aging intensifies, the number of people living with PD is projected to exceed 12 million by 2030, imposing an increasingly heavy socioeconomic burden (2). Gait impairment is one of the most debilitating motor symptoms of PD, characterized by reduced gait speed, shortened stride length, prolonged double support phase, and the hallmark “Freezing of Gait” (FOG). These symptoms, often accompanied by reduced arm swing during ambulation, severely compromise patients’ activities of daily living (ADL) and overall quality of life. Statistics indicate that approximately 80% of PD patients experience gait disturbances during their disease course, with over 50% eventually developing FOG, which remains the primary cause of falls, fractures, and increased hospitalization risk (3, 4).

Although traditional rehabilitation exercises improve motor function in PD to some extent, significant limitations persist. Specifically, conventional training programs are often monotonous, leading to poor patient adherence (5). Moreover, the efficacy of these interventions is highly dependent on the expertise and experience of the therapist (6). The lack of real-time feedback mechanisms also makes precise adjustment of training intensity challenging (7). Furthermore, constrained by the distribution of medical resources, most patients struggle to access continuous and sufficient rehabilitation services (8). In this context, virtual reality (VR) technology offers a promising alternative. By creating simulated environments, VR transforms repetitive gait training into engaging, game-based tasks while allowing for the precise modulation of training parameters. This not only enhances patient engagement but also facilitates the customization of individualized rehabilitation protocols (9).

In recent years, the body of research investigating VR in PD gait rehabilitation has grown steadily. Multiple randomized controlled trials and meta-analyses have demonstrated that VR training can significantly improve gait speed, balance, and dual-task performance. The underlying therapeutic mechanisms may involve the induction of neuroplasticity, activation of the mirror neuron system, and the integration of cognitive-motor dual tasks (10, 11). The application of VR in PD gait rehabilitation has experienced substantial international growth, particularly after 2015, with the annual publication volume expanding from 4 articles in 2008 to 62 articles in 2024 (a total growth rate of 1,450%), demonstrating its emergence as a highly active and expanding research domain.

While a burgeoning body of randomized controlled trials (RCTs) and systematic meta-analyses has thoroughly scrutinized the clinical efficacy and neural mechanisms of VR training in PD gait rehabilitation, these narrative reviews remain inherently limited to micro-level clinical outcomes of isolated cohorts. They inherently lack the capacity to map the macro-structural evolution, thematic shifting velocity, and global collaborative networks of the entire scientific domain over the past decades. Bibliometric analysis, therefore, bridges this critical knowledge gap. By objectively quantifying large-scale metadata, this study transcends simple efficacy validation to uncover the underlying intellectual landscape, identify structural collaborative barriers between Western and Chinese research paradigms, and systematically forecast emerging technological frontiers that discrete clinical trials cannot capture.

Bibliometrics, as a research methodology based on large-scale literature data, can objectively reveal the current status, hotspot distribution, and evolutionary trends within a specific field. CiteSpace and VOSviewer, the mainstream visual analysis tools in this domain, have been widely utilized to map research progress and detect frontiers in medical science (12). While several recent bibliometric studies have mapped out the visual knowledge networks of VR in generic Parkinson’s disease management (13) or evaluated the global research trends surrounding freezing of gait (14, 15), a comprehensive bibliometric study that explicitly isolates VR clinical training modalities for overall gait disorders, while simultaneously providing a cross-cultural comparison between international and Chinese (CNKI) database outputs, remains limited. Therefore, based on the Web of Science, PubMed, and CNKI databases, this study employs CiteSpace and VOSviewer to conduct a visualized analysis of relevant literature from 2008 to 2025. By systematically mapping publication trends, the distribution of research efforts, thematic hotspots, and frontier evolution, the primary objective of this study is to provide a comprehensive global visualization of the domain’s knowledge structure. Concurrently, as a secondary objective, this study delineates the developmental gaps and structural limitations within Chinese research through an international comparative framework, thereby offering tailored strategic recommendations for future regional advancements.

## 2 Materials and methods

### 2.1 Data sources and search strategy

The literature search was conducted on February 23, 2026. Data were retrieved from the China National Knowledge Infrastructure (CNKI), the Web of Science Core Collection [WoSCC, including Science Citation Index Expanded (SCIE) and Social Sciences Citation Index (SSCI)], and PubMed databases, covering the period from 2008 to 2025.

**Figure 1:**
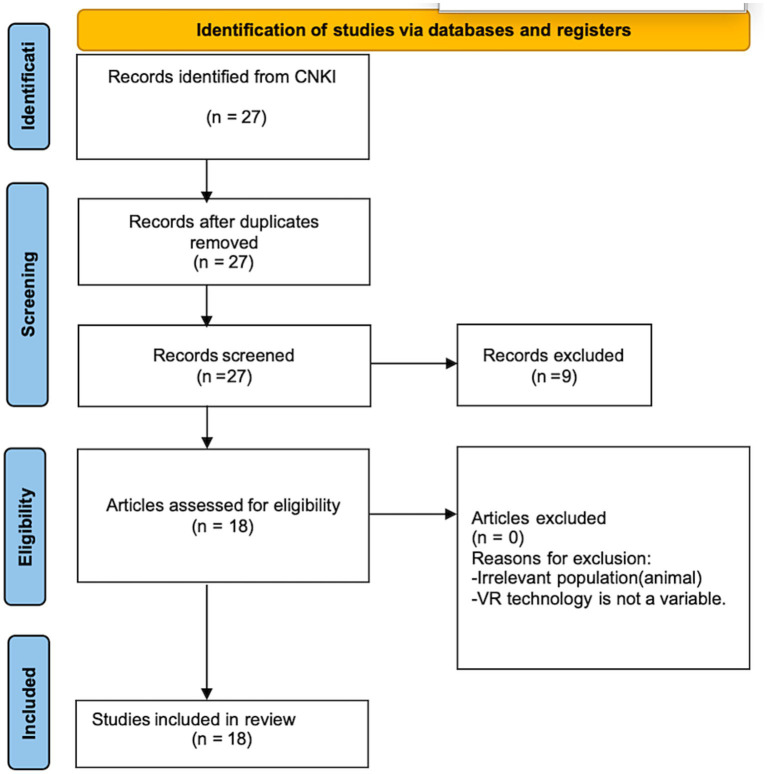
Steps in Chinese literature research.

The search strategy employed for CNKI was as follows: (SU = ‘Parkinson’ OR SU = ‘Parkinson’s disease’ OR TI = ‘Parkinson’ OR TI = ‘Parkinson’s disease’) AND (SU = ‘virtual reality’ OR SU = ‘VR’ OR TI = ‘virtual reality’ OR TI = ‘VR’ OR SU = ‘augmented reality’ OR SU = ‘game-based rehabilitation’) AND (SU = ‘gait’ OR SU = ‘walking’ OR SU = ‘ambulation’ OR SU = ‘freezing of gait’ OR TI = ‘gait’ OR TI = ‘walking’ OR TI = ‘ambulation’ OR TI = ‘freezing of gait’). The source category was restricted to the Chinese Social Sciences Citation Index (CSSCI) and the Chinese Science Citation Database (CSCD). After excluding duplicates and irrelevant records, 18 articles were included. The screening process for Chinese literature is illustrated in Figure 1.

**Figure 2:**
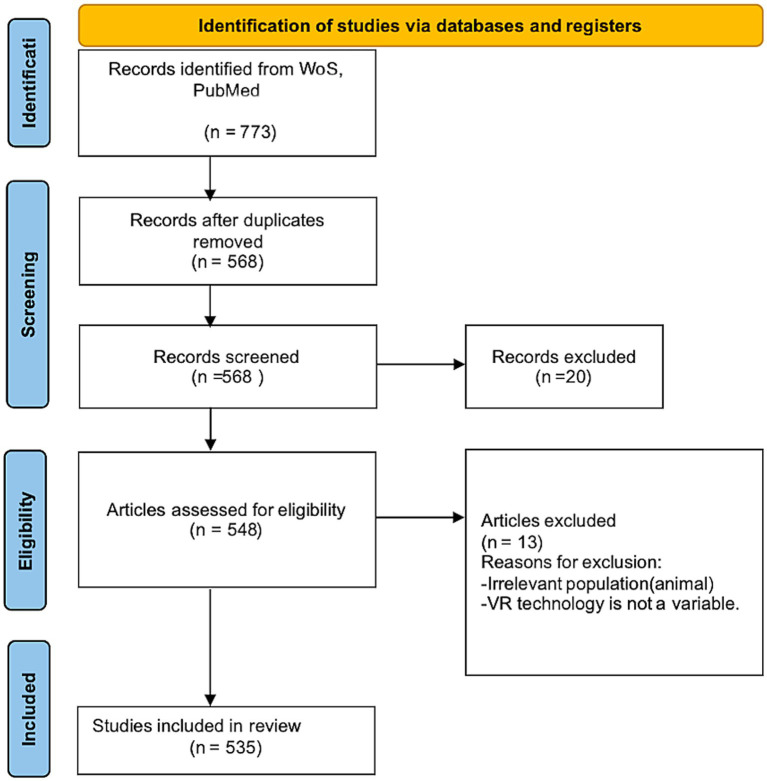
Steps in English literature research.

For the Web of Science (WoS), the topic search string used was: TS = ((“Parkinson* disease” OR “PD” OR “Parkinsonism”) AND (“virtual reality” OR “VR” OR “virtual environment*” OR “immersive virtual reality” OR “exergaming”) AND (“gait” OR “walking” OR “gait disorder*” OR “gait disturbance*” OR “mobility” OR “freezing of gait” OR “FOG” OR “balance”)). Document types were limited to “Article” and “Review,” and the language was restricted to “English.” After removing duplicates, 438 articles were initially identified. For PubMed, the following MeSH terms and Title/Abstract keywords were used: (“Parkinson Disease”[Mesh] OR “Parkinson*”[Title/Abstract] OR “PD”[Title/Abstract]) AND (“Virtual Reality”[Mesh] OR “virtual reality”[Title/Abstract] OR “VR”[Title/Abstract] OR “virtual environment*”[Title/Abstract] OR “immersive virtual reality”[Title/Abstract] OR “head-mounted display”[Title/Abstract] OR “exergaming”[Title/Abstract] OR “virtual training”[Title/Abstract] OR “augmented reality”[Title/Abstract]) AND (“Gait Disorders, Neurologic”[Mesh] OR “gait”[Title/Abstract] OR “walking”[Title/Abstract] OR “gait disturbance*”[Title/Abstract] OR “gait impairment*”[Title/Abstract] OR “gait dysfunction”[Title/Abstract] OR “locomotion”[Title/Abstract] OR “mobility”[Title/Abstract] OR “freezing of gait”[Title/Abstract] OR “FOG”[Title/Abstract] OR “balance”[Title/Abstract] OR “postural control”[Title/Abstract]). The document type was set to “Randomized Controlled Trial,” and the language was restricted to “English.” This search yielded 335 articles. After merging results from both English databases and removing duplicate entries, a final total of 535 English articles were included in the study. The screening process for English literature is shown in Figure 2.

In addition to the overall macro-bibliometric analysis, a subset of specific references (*n* = 40) was selected for in-depth qualitative discussion to interpret the clinical and mechanistic insights of the statistical hotspots. These references were selected based on: (1) landmark articles with high citation frequency or high intermediary centrality within core co-occurrence clusters; (2) representative literature underlying top citation bursts; and (3) recently published high-quality clinical studies detailing emerging trends such as immersive VR and deep learning.

To avoid epistemological distortion, it is critical to clarify the distinct methodological roles assigned to each database in this study. Web of Science (WoS) serves as our primary multi-disciplinary dataset for constructing macro-knowledge graphs, tracking long-term structural shifts, and performing keyword burst detection (including articles and reviews). Concurrently, PubMed was utilized not as a parallel equivalent to WoS, but as a specialized secondary diagnostic pipeline to cross-verify the historical publication velocity specifically within high-evidence clinical randomized controlled trials (RCTs). Because filters in public engines can occasionally be over-inclusive, the higher yield in PubMed reflects broad indexing rather than localized anomalies. Furthermore, the retrieval keyword ‘balance’ was systematically incorporated because dynamic balance control and gait patterns in advanced Parkinson’s disease are neurophysiologically and clinically intertwined; standard clinical assessments (such as the Timed Up and Go test and Berg Balance Scale) invariably evaluate gait kinetics and postural stability synchronously.

### 2.2 Inclusion and exclusion criteria

#### 2.2.1 Inclusion criteria

Studies were included if they met the following criteria: (1) the study population consisted of patients diagnosed with Parkinson’s disease (PD); (2) the intervention involved virtual reality (VR) training; (3) the primary or secondary outcomes included gait-related parameters or gait disorders; (4) the study design was an original research article or a review; (5) the publication language was either Chinese or English; and (6) the study was published between 2008 and 2025.

#### 2.2.2 Exclusion criteria

Studies were excluded based on the following criteria: (1) document types such as conference abstracts, dissertations, theses, or patents; (2) duplicate publications; (3) populations other than PD patients; (4) studies where VR was used solely as an assessment tool rather than an intervention; (5) animal experiments or basic laboratory research; (6) studies that did not report gait-related outcomes; and (7) publications in languages other than Chinese or English.

To ensure theoretical homogeneity, Virtual Reality (VR) was rigorously defined and operationalized based on clinical rehabilitation taxonomies. We explicitly included: (1) Fully immersive systems (Head-Mounted Displays); (2) Semi-immersive or non-immersive digital platforms (screen-based interactive systems); (3) Immersive Augmented Reality (AR) and Mixed Reality (MR) spatial cueing systems; and (4) Technology-driven exergaming modalities. Studies were excluded if the interactive technology did not serve as the primary independent intervention variable for gait or motor training (e.g., descriptive opinion pieces or theoretical engineering hardware designs without clinical participant validation).

### 2.3 Data analysis and visualization

Data analysis and visualization were performed using VOSviewer (version 1.6.20) (16) and CiteSpace (version 6.3.1) (17, 18) within a Java environment, with descriptive statistics managed via Microsoft Excel 2021. Literature from CNKI was exported in RefWorks format, while data from the Web of Science Core Collection (WoSCC) and PubMed were exported as plain text files before being imported into the analysis software. All selected records successfully contained complete bibliographic metadata (including titles, abstracts, keywords, and citation histories), ensuring 100% structural integrity for the algorithmic matrix calculations without any metadata omission.

Web of Science Core Collection (WoSCC) served as the primary database for constructing collaboration and co-occurrence networks, while PubMed and CNKI served as authoritative supplementary channels for clinical and domestic literature. Methodologically, original articles and review articles were processed and synthesized within the bibliometric network. Given that VR training in Parkinson’s disease gait rehabilitation represents a rapidly evolving, technologically dynamic niche, capturing the conceptual keywords and theoretical consolidation from systematic reviews is crucial for mapping the comprehensive intellectual boundaries and capturing early semantic shifting velocity that discrete empirical trials might under-represent.

To ensure analytical precision across these combined datasets, records from WoSCC and PubMed were merged and deduplicated using CiteSpace’s text-processing utility based on digital object identifiers (DOIs) and title matching. Customized thesaurus files were implemented in both software programs to manually standardize synonymous entities: variant author names (e.g., merging “Lewis S” into “Lewis SJ”), institutional sub-departments (e.g., consolidating “Sackler Faculty of Medicine” into “Tel Aviv University”), and geographical regions (e.g., unifying “UK” to “United Kingdom”) were unified. Furthermore, close keyword synonyms (e.g., “virtual reality” and “VR”) were merged, while generic, non-informative automated indexing tags generated by databases (including “humans,” “male,” “female,” “aged,” and “adult”) were systematically masked from the final co-occurrence matrix to prevent baseline demographic noise from obscuring genuine research hotspots.

The specific parameters for CiteSpace were set as follows: a time slicing of 2 years per slice; term sources included titles, abstracts, author keywords, and keywords plus; and node types were sequentially selected as Author, Institution, Country, and Keyword. The selection criteria utilized the *g-index* with a scale factor k = 25. Pruning was performed using Pathfinder and Pruning sliced networks. Betweenness centrality was calculated via the shortest path algorithm, with nodes ≥ 0.1 designated as core bridging hubs based on structural hole theory (18). These bibliometric metrics reflect relative research attention and network connectivity rather than study quality or clinical efficacy. For VOSviewer, the minimum threshold for author analysis was set at 1 publication for Chinese literature and 2 publications for English literature, with a minimum keyword occurrence frequency of 1. Descriptive statistics were managed using Microsoft Excel 2021.

## 3 Results

### 3.1 Analysis of publication volume

**Figure 3:**
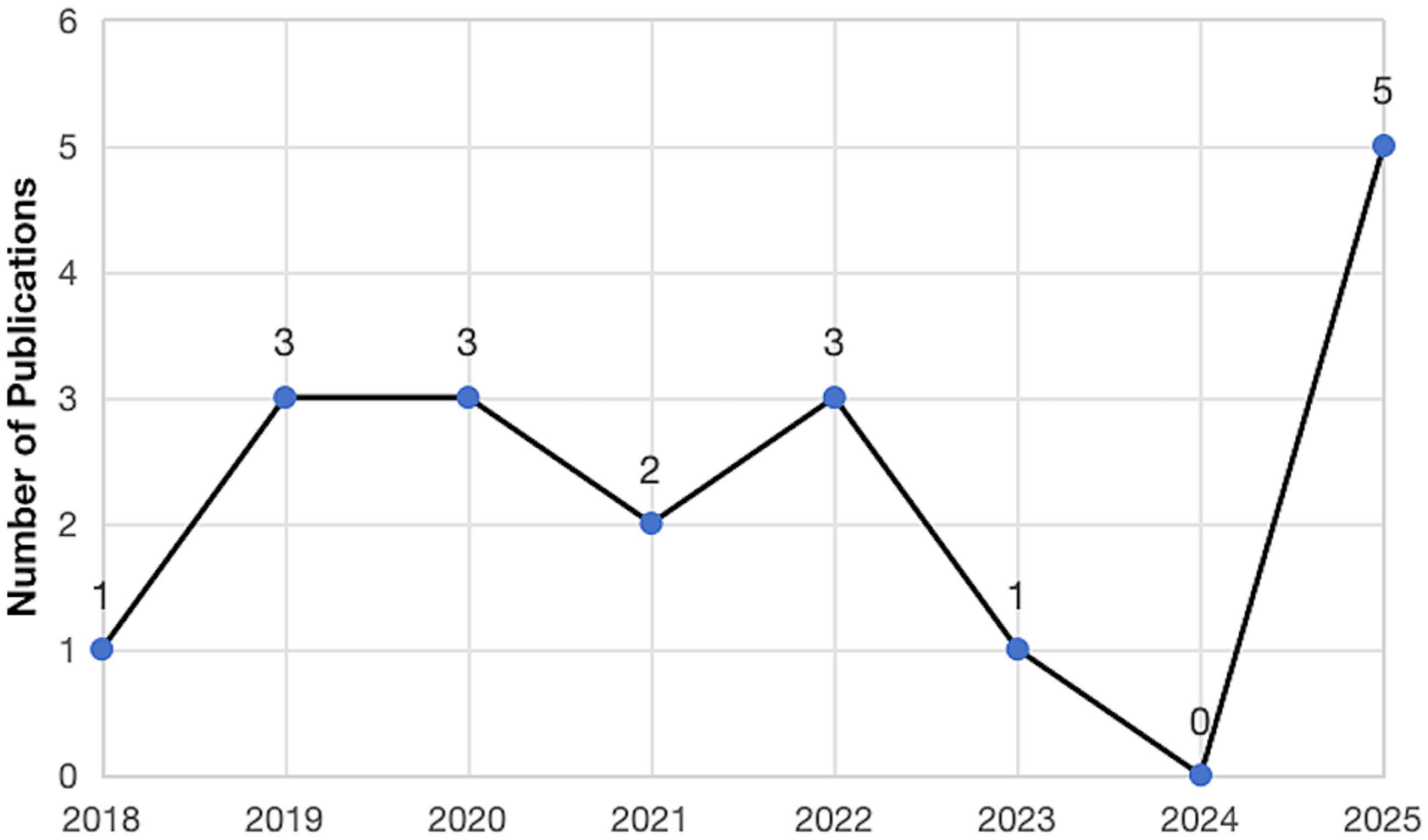
Annual publication volume of Chinese literature.

A total of 18 Chinese articles were identified, published between 2018 and 2025. The overall volume of publications retrieved from the domestic database remains localized, with an average of fewer than three articles per year. Within the parameters of our search strategy, this pattern reflects an alternative, highly focused developmental trajectory for native-language publications (Figure 3).

**Figure 4:**
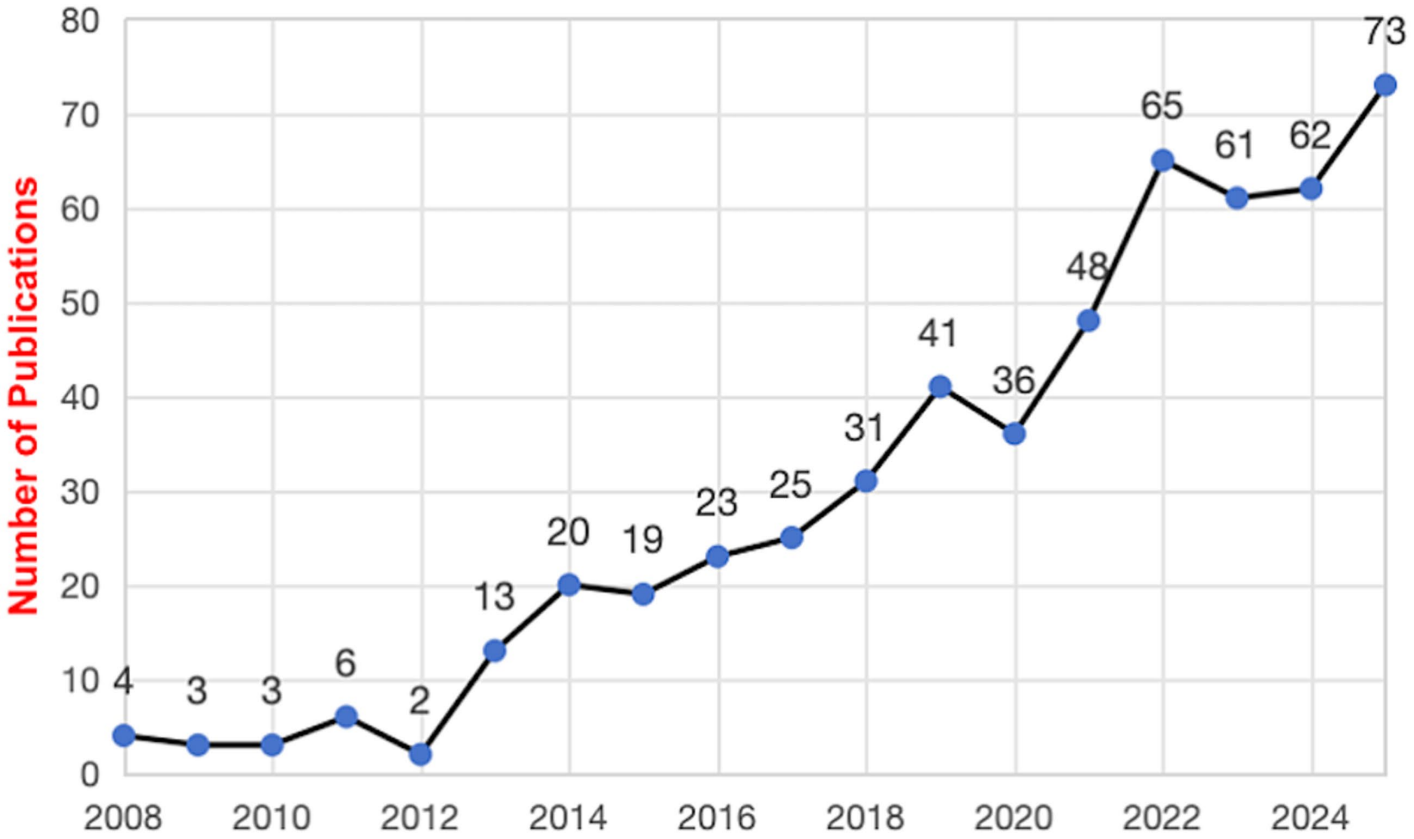
Annual publication volume of English literature.

In contrast, a total of 535 English articles were included. The annual publication volume of English literature exhibited a sustained upward trajectory, with a particularly significant increase beginning in 2015 and reaching a peak between 2020 and 2025. This trend indicates that the application of virtual reality (VR) training in the context of gait rehabilitation for Parkinson’s disease (PD) has become a prominent international research hotspot (Figure 4).

However, when interpreted within the logistically applied database and language parameters, Chinese-language literature accounts for 3.4% of the combined search sample. This variation reflects divergent scales of literature aggregation rather than a baseline quality disparity, highlighting that domestic and international corpuses are currently positioned at distinct structural expansion phases.

### 3.2 Analysis of publication journals

The 18 Chinese articles were distributed across 10 different journals. Chinese Rehabilitation Theory and Practice published two articles, while the remaining journals each published only one. These publications span diverse fields, including rehabilitation medicine, nursing, and biomedical engineering. This distribution indicates that the targeted domestic publications are exploratory and cross-disciplinary, demonstrating a highly diversified initial dissemination across clinical nursing and engineering sectors without clustering around a single dominant journal entity (Table 1).

**Table 1:** Publication volume of Chinese literature by journal.

No.	Journal title	Publications
1	Chinese Rehabilitation Theory and Practice	2
2	Life Science Instruments	1
3	Journal of Nursing Science	1
4	Chinese Journal of Physical Medicine and Rehabilitation	1
5	Chinese Journal of Contemporary Neurology and Neurosurgery	1
6	China Health Standard Management	1
7	Public Communication of Science & Technology	1
8	The Journal of Medical Theory and Practice	1
9	Chinese Acupuncture & Moxibustion	1
10	Zhejiang Medical Journal	1

In contrast, the English literature was primarily published in prestigious international journals within the fields of rehabilitation medicine and neuroscience, such as Neurorehabilitation and Neural Repair, Gait & Posture, and Parkinsonism & Related Disorders. This concentration suggests that the field has a well-established academic exchange platform and a high degree of thematic maturity at the international level.

### 3.3 Analysis of country collaboration networks

**Figure 5:**
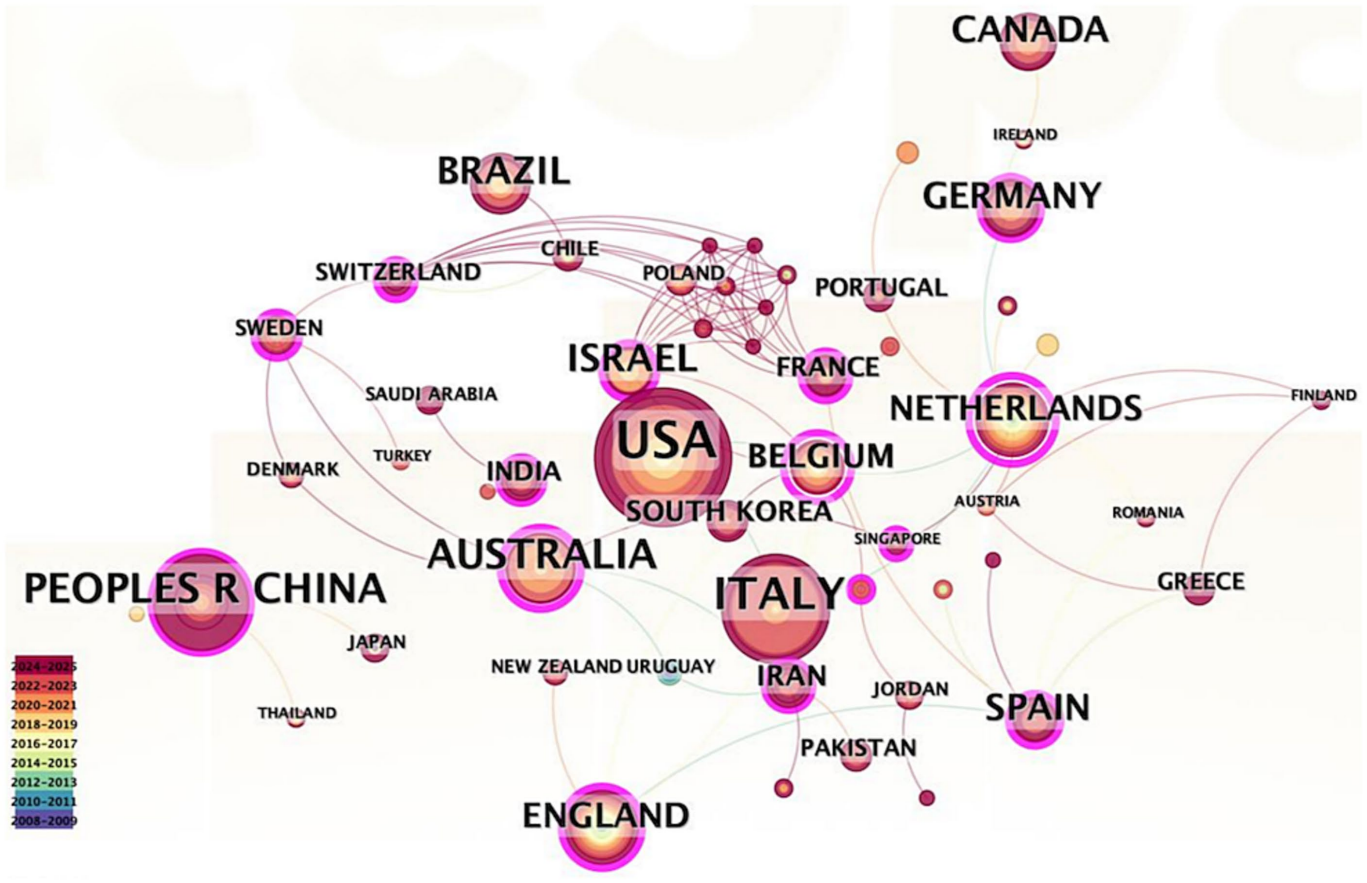
National collaboration network in English literature.

The top five countries by publication volume are the United States (108 papers), Italy (64 papers), China (42 papers), Australia (40 papers), and Brazil (31 papers) (Table 2). The national collaboration network map (Figure 5) reveals that the United States occupies a central position in the network, maintaining close collaborative ties with countries such as Italy, Australia, and Israel. Although China ranks third in total publications within the global English corpus, its collaborative vectors appear relatively localized, as indicated by the dense domestic regional groupings. This structural pattern suggests that while Chinese investigators have established robust independent research pipelines, expanding multi-center cross-linguistic partnerships represents a valuable future evolutionary direction.

**Table 2:** Top 9 countries by publication volume in English literature.

No.	Country	Publications
1	USA	108
2	ITALY	64
3	PEOPLES R CHINA	42
4	AUSTRALIA	40
5	BRAZIL	31
6	ISRAEL	29
7	CANADA	28
8	GERMANY	28
9	SPAIN	27

The top 10 most productive authors are listed in Table 3, with Lewis, Simon J. G. (UK, 24 papers), Mirelman, Anat (Israel, 20 papers), and Hausdorff, Jeffrey M. (USA, 19 papers) ranking as the top three. A transnational research network has emerged among core authors, centered around institutions such as Tel Aviv University (Israel), Radboud University (Netherlands), and KU Leuven (Belgium). This structure highly aligns with the national collaboration network, further confirming the leading positions of these countries and their respective research teams in this field.

**Table 3:** Top 10 most productive authors in the English literature.

No.	Author	Country	Publications
1	Lewis, Simon J. G	UK	24
2	Mirelman, Anat	Israel	20
3	Hausdorff, Jeffrey M.	USA	19
4	Bloem, Bastiaan R.	Netherlands	17
5	Martens, Kaylena A. Ehgotz	Canada	17
6	Shine, James M.	Australia	16
7	Gilat, Moran	Belgium	12
8	Nieuwboer, Alice	Belgium	11
9	Giladi, Nir	Israel	11
10	Pelosin, Elisa	Italy	9

### 3.4 Analysis of research institutions

The distribution of publishing institutions for the Chinese literature is presented in Table 4. The leading institutions include Heilongjiang Provincial Hospital, Beijing Rehabilitation Hospital affiliated with Capital Medical University, and Capital Medical University, each with two publications. The remaining institutions contributed only one paper each, indicating a highly dispersed distribution of research efforts within China.

**Table 4:** Analysis of core contributing research institutions in the Chinese literature.

No.	Institution	Publications
1	Heilongjiang Provincial Hospital	2
2	Beijing Rehabilitation Hospital, Capital Medical University	2
3	Capital Medical University	2

Only institutions with a publication volume of ≥ 2 are displayed. The remaining contributing institutions each contributed a single publication, reflecting a highly fragmented institutional distribution.

**Figure 6:**
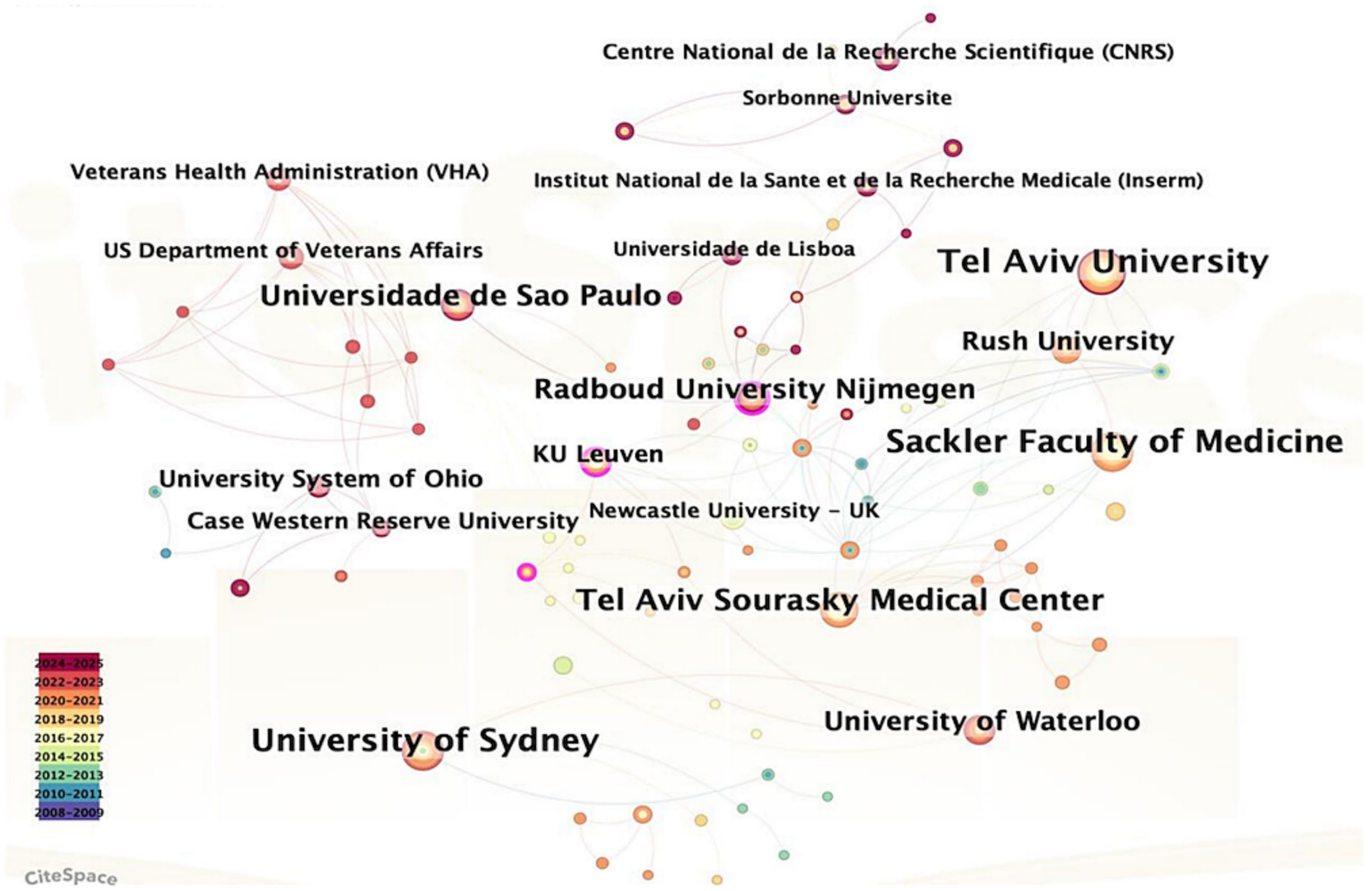
Institutional collaboration network in English literature.

In contrast, the top five institutions in the English literature demonstrate a more centralized research output (Table 5), led by Tel Aviv University (23 papers), followed by the University of Sydney (20 papers), Sackler Faculty of Medicine (20 papers), Tel Aviv Sourasky Medical Center (16 papers), and the Universidade de São Paulo (14 papers). The institutional collaboration network (Figure 6) reveals robust and frequent partnerships among these organizations, which have coalesced into several influential regional research hubs.

**Table 5:** Analysis of research institutions in the English literature.

No.	Institution	Publications
1	Tel Aviv University	23
2	University of Sydney	20
3	Sackler Faculty of Medicine	20
4	Tel Aviv Sourasky Medical Center	16
5	Universidade de São Paulo	14
6	Radboud University Nijmegen	13
7	University of Waterloo	12
8	Rush University	10
9	University System of Ohio	8
10	KU Leuven	8
11	IRCCS Bonino Pulejo	8

### 3.5 Analysis of keywords

High-frequency keywords reflect the core research intensity within a specific field. In bibliometric analysis, a betweenness centrality value of ≥ 0.1 is generally considered to indicate significant influence (19).

As shown in Table 6, after systematically masking baseline database indexing terms to eliminate demographic noise, the intellectual structure of the English literature is strictly defined by core pathological, technological, and functional outcome nodes. The top five keywords ranked by centrality are “virtual reality” (0.42), “Parkinson’s disease” (0.28), “gait” (0.21), “augmented reality” (0.21), and “postural balance” (0.18). Strategically, “virtual reality” marks the primary technological intervention node, exhibiting an outstanding intermediary weight (Centrality = 0.42) that reflects its massive cross-disciplinary connectivity, complemented by the emerging technological frontier of “augmented reality” (Centrality = 0.21). Crucially, functional outcome targets such as “gait” (Centrality = 0.21) and “postural balance” (Centrality = 0.18) display high centrality weights, underscoring their roles as pivotal operational bridges connecting digital therapeutic deployments with objective clinical efficacy verifications.

**Table 6:** Top 10 Keywords in English literature.

No.	Keyword	Centrality	Frequency
1	Parkinson’s disease	0.28	60
2	Virtual reality	0.42	53
3	Gait	0.21	24
4	Augmented reality	0.21	24
5	Postural balance	0.18	18
6	Balance	0.01	16
7	Rehabilitation	0.06	15
8	Gait disorders	0.17	15
9	Cognition	0.17	10
10	Quality of life	0.05	5

#### 3.5.1 Keyword co-occurrence analysis

**Figure 7:**
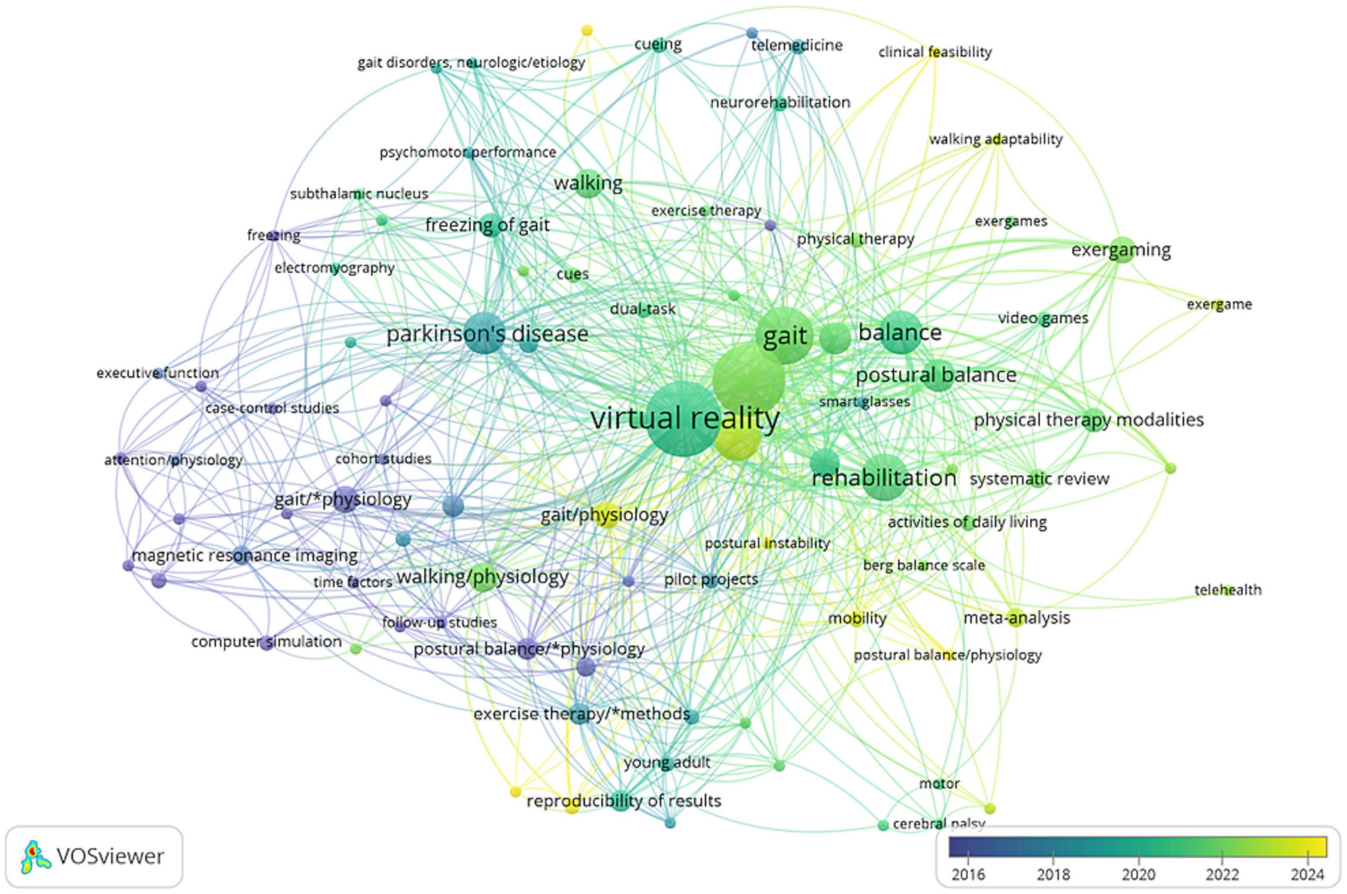
Keyword co-occurrence visualization in English literature.

The co-occurrence network of keywords in the English literature (Figure 7) shows that the top terms by frequency include “virtual reality,” “Parkinson’s disease,” “gait,” and “postural balance.” This distribution indicates that a well-defined research paradigm has been established in the international arena, characterized by the targeted management of neurodegenerative motor impairments (particularly gait and balance deficits) as the primary clinical objective and virtual reality (VR) technology as the core interventional modality.

Keywords in the Chinese literature are primarily concentrated on “virtual reality,” “Parkinson’s disease,” “gait,” “balance,” and “rehabilitation training.” While these core concepts largely overlap with the high-frequency keywords found in the English literature the Chinese-language sample exhibits a specialized path oriented toward traditional clinical exercise frameworks whereas international publications lean heavier into engineering modalities such as “augmented reality.” This thematic divergence indicates that the domestic corpus highly prioritizes the pragmatic implementation and localized verification of baseline clinical rehabilitation outcomes over rapid digital technology iterations

#### 3.5.2 Keyword clustering analysis

**Figure 8:**
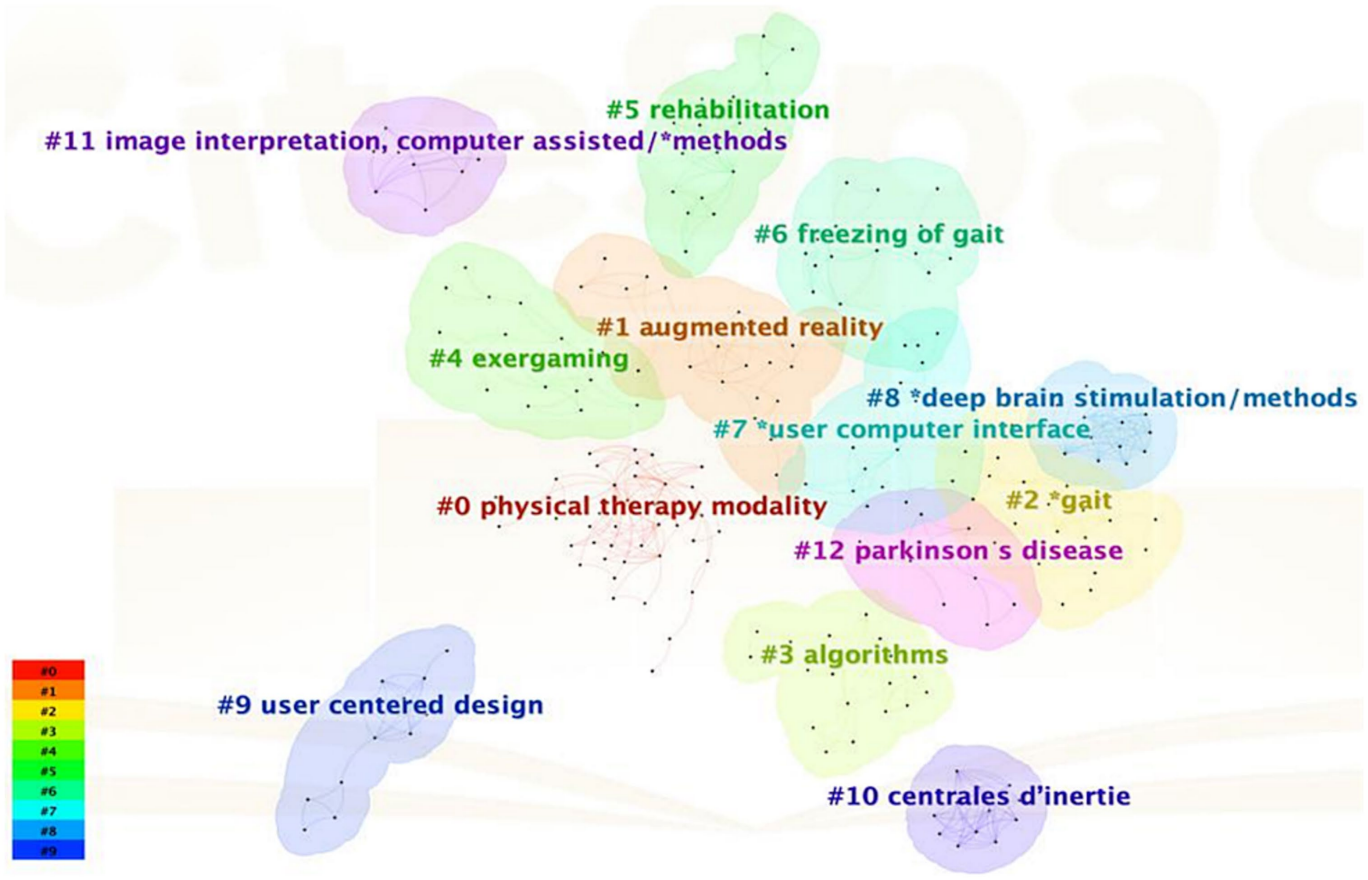
Cluster analysis of keywords in English literature.

Keyword clustering was performed using the Log-Likelihood Ratio (LLR) algorithm in CiteSpace for both Chinese and English literature (20). For the English literature the clustering map (Figure 8) yielded a Modularity Q value of 0.7974 and a Mean Silhouette S value of 0.9387. In bibliometric analysis a Q value > 0.3 indicates a significant community structure while an S value > 0.5 suggests that the clustering is generally considered reasonable and consistent (21).

The clustering map for the English literature generated a total of 13 clusters (#0 to #12). Each cluster exhibited a silhouette value greater than 0.85, confirming high reliability and internal consistency of the results. The primary clusters and their corresponding core research themes are detailed in Table 7, encompassing directions such as: Physical therapy modalities (#0), Augmented reality (#1), Gait and brain mechanisms (#2), Freezing of gait (#6) and User-centered design (#9).

**Table 7:** Information on 13 representative keyword clusters in English literature.

Cluster ID	Size (nodes)	Silhouette (S)	Major keywords (LLR)
#0	42	0.919	Physical therapy modality, *parkinson disease, motivation, exergaming, postural instability
#1	23	0.931	Augmented reality, *augmented reality, *parkinson disease, walking speed/physiology, *parkinson disease/physiopathology
#2	21	0.899	*Gait, psychomotor performance, brain mapping, motor activity, neuropsychological tests
#3	19	0.888	Algorithms, electroencephalography, exercise therapy/*methods, normal distribution, *movement disorders
#4	19	0.926	Exergaming, digital technology, *parkinson disease/diagnosis/therapy, *parkinson disease/therapy, *stroke/therapy
#5	18	1	Rehabilitation, postural balance/physiology, cognition, clinical trials, *stroke rehabilitation/methods
#6	17	0.873	Freezing of gait; electromyography, deep brain stimulation, subthalamic nucleus, gait disorder
#7	16	0.976	*User computer interface, biomechanical phenomena, task performance and analysis, reaction time, arm/*physiopathology
#8	14	0.988	*Deep brain stimulation/methods, *brain mapping, *gait disorders, neurologic/etiology/therapy, *parkinson disease/complications/therapy, basal ganglia/*physiopathology
#9	12	1	User centered design, prototypes, cues, inclusive design, sensory cues
#10	12	0.992	Centrales d’inertie visual biofeedback, suppléance sensorielle, orientation posturale, *feedback, sensory
#11	9	1	Image interpretation, computer assisted/*methods, magnetic resonance imaging/*methods, computer systems, brain mapping/*methods, evoked potentials/*physiology
#12	8	0.921	Parkinson s disease, rehabilitation medicine, cross over study, amyotrophic lateral sclerosis – frontotemporal spectrum disorder, multiple sclerosis

The high silhouette values across these clusters reflect a clear separation of research topics, ranging from fundamental physiological mechanisms to innovative technological interventions and patient-focused design.

#### 3.5.3 Keyword burst analysis

Keyword burst detection was performed using CiteSpace to identify terms that experienced a sudden surge in citation frequency within a short period, which serves as an indicator of emerging research trends (22).

**Figure 9:**
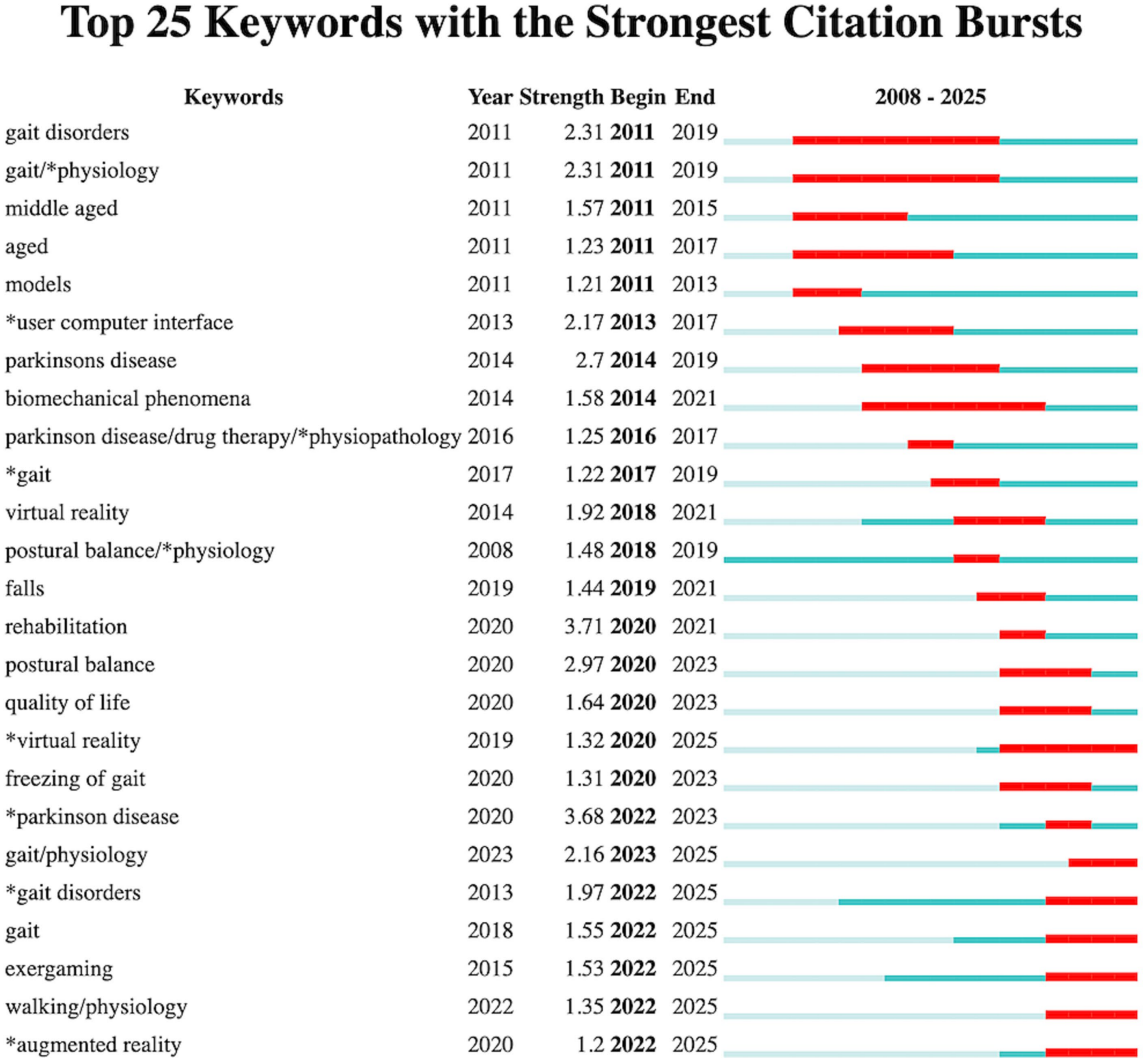
Top keywords with the strongest citation bursts in English literature.

The keyword burst map for the English literature (Figure 9) reveals several highly influential terms with significant burst strength. In the earlier stages, the domain was dominated by generic clinical concepts and patient demographics, such as “gait disorders” (Strength = 2.31, 2011–2019), “middle aged” (Strength = 1.57, 2011–2015), and “*user computer interface” (Strength = 2.17, 2013–2017). Mid-term bursts saw a narrower tactical focus on clinical phenomena like “postural balance” (Strength = 2.97, 2020–2023) and “freezing of gait” (Strength = 1.31, 2020–2023). This evolutionary trajectory indicates a clear shift in the research frontier: moving from foundational investigations into generic clinical concepts toward precise technological diversification and physiological quantification. As illustrated in Figure 9, the frontline extending through 2025 is mathematically dominated by a high-strength burst cluster focused on specific technological modalities and objective indicators. This includes expanding computational and immersive paradigms like “exergaming” (Strength = 1.53, 2022–2025) and “augmented reality” (Strength = 1.20, 2022–2025), closely coupled with a strict focus on quantifiable biophysical outcomes such as “gait/physiology” (Strength = 2.16, 2023–2025) and “walking/physiology” (Strength = 1.35, 2022–2025). This algorithmic clustering demonstrates that the current research frontier is prioritizing the optimization of VR interventions through data-driven, gamified, and patient-specific physiological monitoring.

## 4 Discussion

### 4.1 General trends in virtual reality training for gait disorders in Parkinson’s disease

Through a visualized analysis of domestic and international literature from 2008 to 2025, this study delineates the current research landscape and developmental networks of virtual reality (VR) training within the field of gait rehabilitation for Parkinson’s disease (PD). Regarding publication volume, English-language literature has exhibited a sustained and rapid growth trajectory. This upward trend aligns with the publication of several recent systematic reviews, reflecting the increasing global academic attention toward the clinical utility of VR technology in PD rehabilitation (23).

From the perspective of semantic cluster mapping, the clinical enthusiasm for VR-based rehabilitation is fundamentally rooted in its neuroplastic induction effects (24), a foundational theme that underpins early high-centrality literature. Research suggests that repetitive, task-oriented training, combined with real-time feedback, can enhance synaptic plasticity within the motor and premotor cortices, thereby optimizing motor output. The activation of the mirror neuron system (25) is considered a key underlying mechanism; specifically, the observation and execution of actions within a virtual environment can stimulate the cerebellum, as well as cortical and subcortical regions associated with motor control, subsequently invigorating the sensorimotor system. Furthermore, by integrating cognitive tasks with dual-task training, VR interventions can facilitate improvements in executive function and attention (26), which are critical for effective gait control in the daily lives of patients with PD. The substantial publication density surrounding these biological pathways validates why generic clinical classifications initially dominated our keyword timeline.

Notably, while research into the motor symptoms of PD has reached a degree of maturity, the application of VR technology in gait rehabilitation continues to evolve and deepen, moving from foundational mechanistical investigations into technological diversification and physiological quantification. This evolutionary trajectory is empirically validated by our keyword burst analysis (Figure 9). In the earlier stages, the domain was structurally anchored by generic clinical classifications, such as “gait disorders” (2011–2019). Mid-term developments witnessed a deep tactical refinement targeting specific clinical syndrome clusters, highlighted by the bursts of “postural balance” (2020–2023) and “freezing of gait” (2020–2023). More recently, the ongoing bursts extending through 2025 are mathematically dominated by specific interactive modalities, such as “exergaming” (Strength = 1.53) and “augmented reality” (Strength = 1.20), closely coupled with a strict clinical focus on quantifiable biophysical outcomes such as “gait/physiology” (Strength = 2.16) and “walking/physiolog” (Strength = 1.35). This algorithmic shifting pattern demonstrates that the academic frontline has evolved past simple efficacy observation toward data-driven, gamified, and precision-monitored rehabilitation strategies.

### 4.2 Distribution characteristics of research strength

#### 4.2.1 Analysis of national collaboration networks

The national collaboration network reveals that the United States occupies a pivotal position, leading in publication volume and maintaining a high-density collaborative network with countries such as Italy, Australia, and Israel. This dominance is closely linked to these nations’ long-standing expertise in neurorehabilitation and their early strategic focus on emerging technological modalities.

Notably, while China ranks third in total publications (42 papers), the collaboration map indicates that within the parameters of the analyzed international databases, the cooperative networks of Chinese investigators appear primarily localized and regionally clustered. This structural configuration suggests that while independent domestic research pipelines have achieved substantial scale, establishing deeper cross-linguistic and trans-national partnerships represents a vital next phase to integrate these native strengths into the broader global ecosystem. To amplify China’s global research footprint, domestic teams should prioritize integration into the global research ecosystem through participation in multi-center clinical trials and the co-development of international standardized databases.

#### 4.2.2 Characteristics of author and institutional collaboration

The collaboration network of English-language literature is characterized by highly cohesive, expert-centered clusters. Key researchers, including Lewis, Simon J. G. (24 papers), Mirelman, Anat (20 papers), and Hausdorff, Jeffrey M. (19 papers), have established a robust transnational network centered around elite institutions such as Tel Aviv University (Israel), Radboud University (Netherlands), and KU Leuven (Belgium). Such structured consortia facilitate the pooling of research resources, the standardization of protocols, and the execution of high-impact multi-center studies, thereby driving the field’s advancement.

Institutional analysis further confirms this concentration of expertise, with Tel Aviv University (23 papers), the University of Sydney (20 papers), and the Sackler Faculty of Medicine (20 papers) acting as central hubs within the collaborative landscape.

In contrast, the tracked domestic research community exhibits alternative organizational features, characteristically driven by exploratory, multi-center, or individual clinical trials that have not yet coalesced into a single dominant institutional alliance. This structural variation—reflecting a highly diversified and decentralized initial dissemination phase across local clinical centers—underscores a divergent developmental stage compared to the long-standing international consortia, suggesting that the domestic field is currently prioritizing localized clinical validation over centralized academic clustering. Our finding that the USA spearheads international networks and serves as a primary hub for global collaborations aligns tightly with the baseline bibliometric trends reported by Wu et al. (13) regarding overall virtual reality interventions in Parkinson’s disease management. However, while Wu et al. provided a macro-overview of general PD rehabilitation, our dual-database comparative analysis goes a step further by revealing that Chinese domestic research within the CNKI database remains structurally fragmented and underrepresented in these global networks, highlighting a critical geographical bottleneck in international knowledge exchange.

### 4.3 Research hotspots and progress

#### 4.3.1 Analysis of research hotspots

Keyword co-occurrence analysis reveals that high-frequency terms in the English literature include “parkinson’s disease” (60 instances) “virtual reality” (53 instances) “gait” (24 instances) “augmented reality” (24 instances) and “postural balance” (18 instances). These findings reflect a primary research focus on the application of VR technology in rehabilitating motor functions specifically gait and balance. Centrality analysis identifies nodes such as “virtualreality” (0.42) “parkinson disease” (0.28) and “postural balance” (0.18) as critical bridging components that integrate diverse research themes within the co-occurrence network.

Notably, “augmented reality” (AR) has emerged as a significant keyword (24 instances), suggesting that researchers are increasingly exploring interventional modalities with higher ecological validity than conventional VR. AR technology, which overlays virtual information onto natural environments, offers unique advantages for gait training within real-world daily activity scenarios (27). In contrast, Chinese keywords demonstrated a distinct thematic distribution predominantly focused on classic clinical rehabilitation paradigms. This thematic variance should be interpreted strictly within the logistical constraints of cross-database indexing variations and language restrictions. Rather than a baseline chronological gap, it underscores a pragmatic, application-driven path wherein domestic research highly prioritizes immediate clinical implementation and localized efficacy verifications over abstract digital engineering iterations.

#### 4.3.2 Clustering structure of research themes

Thirteen distinct clusters were identified in the English literature (Figure 8), which can be categorized into five major research directions:

Integration of Physical Therapy and Exergaming (#0): This cluster emphasizes keywords such as “physical therapy modality,” “motivation,” and “exergaming.” It reflects a trend of combining traditional physiotherapy with gamification elements to enhance treatment adherence and therapeutic outcomes in PD patients. Augmented Reality and Technological Integration (#1): Focusing on AR applications, this direction also explores the underlying pathophysiological mechanisms of PD, demonstrating an integrated approach to technology and clinical theory. Gait and Brain Mechanisms (#2, #6, #8): These clusters investigate the neurological foundations of gait, involving brain mapping, deep brain stimulation (DBS), and the basal ganglia. Evidence suggests that VR training promotes neuroplasticity by activating specific brain regions, thereby optimizing gait control (28). User-Centered Design (#9): This cluster centers on “user-centered design,” “cues,” and “inclusive design.” It embodies a patient-oriented philosophy, reflecting the global development toward tailored, individualized rehabilitation strategies and customized clinical protocols (29). Comparative Studies Across Multiple Diseases (#12): This direction involves comparative research between PD and other neurodegenerative disorders (e.g., amyotrophic lateral sclerosis and multiple sclerosis), exploring the generalizability and specificity of VR interventions.

Conversely, the analyzed Chinese-language literature exhibits a highly generalized and non-overlapping structural layout. Under the current strict filtering criteria, the themes manifest as independent clinical verifications, reflecting an exploratory and clinical-first orientation that currently emphasizes field-based treatment outcomes rather than the mathematical clustering of theoretical neurobiological mechanisms.

#### 4.3.3 Evolutionary characteristics of research Frontiers

Keyword burst analysis delineates the chronological evolution of the research frontier. The early stage was characterized by macro-level clinical positioning capturing “gait disorders” and general human computer interaction platforms. The middle stage witnessed a deep tactical refinement targeting specific syndrome clusters highlighted by the bursts of “postural balance” and “freezing of gait.” The recent stage (extending through 2025) reveals two prominent, synergistic trends

##### 4.3.3.1 Integration of active technological modalities

The sustained bursts of “exergaming” and “augmented reality” demonstrate that clinical research has advanced past simple treadmill interventions into immersive, game-based, and highly interactive digital paradigms to boost adherence.

##### 4.3.3.2 Deepening of biophysical and physiological quantification

The strong, ongoing bursts of “gait/physiology” and “walking/physiology” mathematically signify a paradigm shift toward data-driven, sensor-compatible, and objectively monitored precision outcomes.

### 4.4 Focused analysis of research frontiers

It must be explicitly stated that the specific clinical dose–response dynamics and optimal VR training parameters discussed below are systematically synthesized from secondary clinical evidence (such as reference meta-analyses) rather than derived directly from our bibliometric software algorithms. Bibliometric evolutionary trends indicate that the research frontier is actively pivoting from foundational efficacy observations to structured programmatic parameters. Recent high-yield meta-analyses by Wu and Zhang (30) and Yu et al. (31) heavily populate this frontier by defining empirical thresholds for training frequency, session duration, and disease-duration benefits, thereby supplying an evidence-based foundation to supplement our structural mapping.

This paradigm shift toward precision rehabilitation is tightly coupled with technological diversification within global metadata. As structurally integrated in our content analysis (Table 8), recent clinical trials exhibit a prominent architectural divergence based on VR immersion levels (32, 33). In close alignment with recent high-strength keyword bursts (“gait/physiology,” “exergaming”) and independent domain tracking by Jiao et al. (14) and Qi et al. (15), the choice of immersion level has emerged as a central thematic axis heavily associated with reported improvements in Freezing of Gait (FOG) frequency and dual-task performance. While high-immersion systems are valued for their intensive visual cueing properties to suppress FOG, non-immersive digital platforms remain heavily investigated for home-based protocols due to superior patient adherence and lower technical barriers. This systemic divergence mirrors ongoing controversies in active sub-clusters regarding Specific VR versus Non-specific VR, where therapeutic efficacy correlates more with content design than technological format (10). Driven by these sub-clusters, the global frontier is accelerating toward multimodal integration—combining VR with wearable sensors, EEG, and fNIRS—to monitor real-time biophysical outcomes, a trajectory validated by high-centrality network nodes (34, 35). Consequently, the dual-task paradigm has emerged as a core thematic hub (Cluster #2), leveraging immersive environments to optimize transfer effects under ecological cognitive loads (36).

**Table 8:** Intervention protocols and therapeutic effects of different VR modalities on Freezing of Gait (FOG).

VR modality	Representative devices/Forms	Frequency and duration	Visual cue types	Primary improvements in FOG	Sources (references)
Immersive VR	Head-mounted displays (HMDs), e.g., Oculus Rift, HTC Vive	20–30 min/ session, 3–5 sessions/ week, for 4–8 weeks	Gait-triggered visual cues, virtual obstacles, dynamic scene navigation	Significant reduction in FOG episodes; improved TUG dual-task performance; enhanced patient-reported walking confidence	Wu and Zhang (30); Ishaq et al. (33)
Non-immersive VR	Screen projection + motion sensors (e.g., Microsoft Kinect, Nintendo Wii)	30–45 min/ session, 2–3 sessions/ week, for 6–12 weeks	Gait trajectory guidance, rhythmic visual cues, floor-projected cues	Improvements in gait speed, stride length, and gait variability; smaller reduction in FOG frequency but higher home-based compliance	Wu and Zhang (30); Lheureux et al. (10); Yau et al. (34)

### 4.5 Limitations and future perspectives

This study has several inherent methodological limitations that warrant acknowledgment. First, while our broad search string captured the core domain of virtual reality, some highly specific, granular technical terms (e.g., “exergaming,” “mixed reality,” or specific devices like “Kinect”) and closely correlated clinical balance complications (e.g., “falls” or “postural instability”) were not explicitly enumerated in the secondary search strings, which may theoretically underestimate certain specialized technical niches. Second, the fundamental structural differences between WoS (global metadata) and PubMed (filtered clinical RCTs) preclude direct mathematical comparison, introducing a database selection bias. Third, our strict selection criteria within the Chinese database (CNKI), which was restricted to CSSCI and CSCD core journals to ensure higher editorial and methodological quality, inevitably omitted a substantial volume of lower-tier regional or clinical implementation literature. This specific filtering mechanism likely contributed to the significant underestimation of the total volume of Chinese research outputs compared to international datasets.

Future research should focus on the following directions: (1) Conducting large-scale, multi-center randomized controlled trials (RCTs) to establish a robust evidence-based hierarchy. (2) Systematically exploring optimal training parameters to transition from experience-based therapy to precision rehabilitation. (3) Utilizing advanced neuroimaging (e.g., fMRI, fNIRS) to elucidate the neuroplastic mechanisms of motor network reorganization induced by VR. (4) Accelerating the synergy between VR and wearable devices, artificial intelligence, and telemedicine to provide personalized gait management for patients with Parkinson’s disease.

### 4.6 Implications for physiotherapy practice

Closely aligned with the dual-task and user-centered design paradigms identified in Cluster #2 and Cluster #9, the translated evidence from global metadata indicates that clinical heterogeneity significantly shapes practical physiotherapy implementations. Specifically, the literature reveals that the Hoehn-Yahr (H&Y) stage serves as a critical source of this heterogeneity, partitioning how patients interact with mapped VR modalities. High-yield clinical trials compiled in these sub-clusters demonstrate that early-stage patients (H&Y 1–2.5) retain superior cognitive reserves, yielding optimal adherence and pronounced gains in dual-task gait speed and stride length. Conversely, while middle-to-late-stage cohorts (H&Y 3–4) show potential tracking in FOG suppression, their systemic tolerance for high-immersion systems significantly declines due to bibliometrically documented risks of cybersickness (37) and elevated fatigue-related dropouts. Consequently, integrating our user-centered clusters (#9) into practice implies that physiotherapy protocols (38) must be dynamically optimized based on disease stages: early-stage interventions should prioritize high-immersion formats to maximize neuroplastic induction, whereas advanced stages are better suited for non-immersive adaptations that safeguard training sustainability.

## 5 Conclusion

This study utilized VOSviewer and CiteSpace to systematically map the research landscape of virtual reality (VR) training for gait disorders in Parkinson’s disease from 2008 to 2025. The results demonstrate that VR-based gait rehabilitation has become a significant international research hotspot with a sustained increase in publication volume. While a robust global collaboration network—centered in the United States, Italy, and Israel—has been established, research in China remains in its nascent stage, characterized by fragmented efforts and limited international integration.

The research frontier has evolved from foundational efficacy validation toward an intelligent, remote, and personalized paradigm. Current hotspots emphasize the optimization of intervention parameters, the application of immersive VR, and the exploration of underlying neural mechanisms through multimodal integration. To bridge the gap between domestic and international research, future efforts in China should focus on strengthening global collaboration, conducting high-quality multi-center trials, and leveraging emerging technologies to provide precise, individualized gait interventions for the growing PD population.

In conclusion, bibliometric and content analyses demonstrate that VR integration into PD gait protocols has evolved from an entry-level exploratory tool into a highly structured, rapidly expanding global research domain with significant developmental velocity.

## Data Availability

The original contributions presented in the study are included in the article/supplementary material, further inquiries can be directed to the corresponding author.
